# Supplementary material for: Risk factors associated with gall bladder cancer in high incidence areas in India: a systematic review protocol
Source: BMJ Open. 2022 Mar 1;12(3):e056849. doi: 10.1136/bmjopen-2021-056849 (PMC8889324; doi:10.1136/bmjopen-2021-056849)
Supplement: Supplementary data [file bmjopen-2021-056849supp001.pdf]

Review title: Risk factors associated with gall bladder cancer in high incident areas in India
Supplementary file:1
PCC Statement: Population: Adults (>18 years) with confirmed gall bladder cancer
Concept: Risk factors (exposures)
Context: High incidence areas in India defined as the North, East and North-East India, comprising the Ganga-Brahmaputra belt.
Search strategy used for each database:

1. **PUBMED**

#1-Domain 1
"gallbladder cancer*"[All Fields] OR "Gall bladder cancer"[All Fields] OR "Gallbladder neoplasms"[MeSH Terms] OR "gall bladder neoplasm*"[All Fields] OR "gallbladder carcinoma*"[All Fields] OR "gall bladder carcinoma*"[All Fields] OR "Carcinoma of gallbladder"[All Fields] OR "gallbladder tumor*"[All Fields] OR "gall bladder tumor*"[All Fields] OR "Cancer of gallbladder"[All Fields]
#2-Domain 2
"risk factor*"[All Fields] OR "epidemiological factor*"[All Fields] OR "associate"[All Fields] OR "associated"[All Fields] OR "associates"[All Fields] OR "associating"[All Fields] OR "association"[MeSH Terms] OR "association"[All Fields] OR "associations"[All Fields] OR "correlat*"[All Fields] OR "relation*"[All Fields]
#3- Domain 3
"india"[MeSH Terms] OR "india"[All Fields] OR "india s"[All Fields] OR "indias"[All Fields]
#1 AND #2 AND #3
Final Search
("gallbladder cancer*"[All Fields] OR "Gall bladder cancer"[All Fields] OR "gallbladder neoplasms"[MeSH Terms] OR "gall bladder neoplasm*"[All Fields] OR "gallbladder carcinoma*"[All Fields] OR "gall bladder carcinoma*"[All Fields] OR "Carcinoma of gallbladder"[All Fields] OR "gallbladder tumor*"[All Fields] OR "gall bladder tumor*"[All Fields] OR "Cancer of gallbladder"[All Fields]) AND ("risk factor*"[All Fields] OR "epidemiological factor*"[All Fields] OR "correlat*"[All Fields] OR "associat*"[All Fields] OR "relation*"[All Fields] OR "association"[MeSH Terms]) AND ("india"[MeSH Terms] OR "india"[All Fields] OR "india s"[All Fields] OR "indias"[All Fields])"associating"[All Fields] OR "association"[MeSH Terms] OR "association"[All Fields] OR "associations"[All Fields] OR "correlat*"[All Fields] OR "relation*"[All Fields])
Total number of titles: 384

2. **EMBASE**

Domain 1:
("gallbladder cancer*" or "Gall bladder cancer" or "Gallbladder neoplasms" or "gall bladder neoplasm*" or "gallbladder carcinoma*" or "gall bladder carcinoma*" or "Carcinoma of gallbladder" or "gallbladder tumor*"or "gall bladder tumor*" or "Cancer of gallbladder").af.
Domain 2:
('risk factor*' or 'epidemiological factor*' or correlat* or relation* or associat*).af
Domain 3:
(india* or India*).af.
Final search:
1 and 2 and 3
Number of titles: 942
Set filters to English language, human and year1990 onwards
Total number of titles: 885

3. **Web of Science**

Domain 1
((((((((((ALL=("gallbladder cancer*")) OR ALL=("Gall bladder cancer"))) OR ALL=("Gallbladder neoplasms")) OR ALL=("gall bladder neoplasm*")) OR ALL=("gallbladder carcinoma*")) OR ALL=("gall bladder carcinoma*")) OR ALL=("Carcinoma of gallbladder")) OR ALL=("gallbladder tumor*")) OR ALL=("gall bladder tumor*")) OR ALL=("Cancer of gallbladder")
Domain 2
((((ALL=("risk factor*")) OR ALL=("epidemiological factor*")) OR ALL=("associat*")) OR ALL=(correlat*)) OR ALL=(relation*)
Domain 3
(ALL=(india*)) OR ALL=(India*)
Final search:
((#1) AND #2) AND #3
Total number of titles: 373

4. **SCOPUS**

Domain 1
"gallbladder AND *cancer*"* OR *"Gall bladder cancer"* OR *"Gallbladder neoplasms"* OR *"gall bladder ne OR oplasm*"* OR *"gallbladder carcinoma*"* OR *"gall bladder carcinoma*"* OR *"Carcinoma of gallbladder"* OR *"gallbladder tumor*"* OR *"gall bladder tumor*"* OR *"Cancer of gallbladder"*
Domain 2
'risk AND *factor*'* OR *epidemiological* AND *factor*'* OR *correlat** OR *relation** OR *associat ** Domain 3
*india** OR *india**

Final search:
(*'risk* AND *factor*'* OR *'epidemiological* AND *factor*'* OR *correlat** OR *relation** OR *associ at**) AND (*"gallbladder* AND *cancer*"* OR *"Gall bladder cancer"* OR *"Gallbladder neoplasms"* OR *"gall bladder ne OR oplasm*"* OR *"gallbladder carcinoma*"* OR *"gall bladder carcinoma*"* OR *"Carcinoma of gallbladder"* OR *"gallbladder tumor*"* OR *"gall bladder tumor*"* OR *"Cancer of gallbladder"**) AND (*india** OR *india**) AND (EXCLUDE (PUBYEAR , *1989*) OR EXCLU DE (PUBYEAR , *1980*) OR EXCLUDE (PUBYEAR , *1978*) OR EXCLUDE (PUBYEAR , *1976*))
Total number of titles: 212

5. **CINAHL plus (EBSCO)**

Domain 1
"gallbladder AND cancer*" OR "Gall bladder cancer" OR "Gallbladder neoplasms" OR "gall bladder ne OR oplasm*" OR "gallbladder carcinoma*" OR "gall bladder carcinoma*" OR "Carcinoma of gallbladder" OR "gallbladder tumor*" OR "gall bladder tumor*" OR "Cancer of gallbladder"
Domain 2
'risk AND factor*' OR 'epidemiological AND factor*' OR correlat* OR relation* OR associat*
Domain 3
india* OR India*
Final search:
S1 AND S2 AND S3
Total number of titles: 36

6. **Open Grey**

(''gallbladder AND cancer*'' OR "Gall bladder cancer" OR "Gallbladder neoplasms" OR "gall bladder ne OR oplasm*" OR "gallbladder carcinoma*" OR "gall bladder carcinoma*" OR "Carcinoma of gallbladder" OR "gallbladder tumor*" OR "gall bladder tumor*" OR "Cancer of gallbladder") AND ('risk AND factor*' OR 'epidemiological AND factor*' OR correlat* OR relation* OR associat*) AND india* OR India*

Total number of title = 1

7. **GoogleScholar**

(''gallbladder cancer'' OR "Gall bladder cancer" OR "Gallbladder neoplasms" OR "gall bladder neoplasm" OR "gallbladder carcinoma" OR "gall bladder carcinoma" OR "Carcinoma of gallbladder" OR "gallbladder tumor" OR "gall bladder tumor" OR "Cancer of gallbladder") AND ('risk facto" OR epidemiological OR factor OR correlation OR relation OR relationship OR association OR associated) AND India

Number of titles = 146

Added filter for articles between 1990-2021

Total number of titles = 93

Inclusion criteria:

Studies

1. Reporting association (positive or negative or no) with at-least one risk factors for gallbladder cancer mortality, incidence, hazards, survival, causation
2. Conducted among India patients, residing in India
3. Results based on biospecimens only from Indian patients residing in India
4. Conducted after year 1990
5. limited to English language
6. Studies not mentioning 'India' in their title or abstract but study setting indicative of geographical region within India

Exclusion criteria:

Studies

1. Measuring only prevalence/incidence of gallbladder cancer
2. Experimental studies measuring efficacy of therapeutic/surgical interventions
3. Conducted on Indian population* living outside India
4. Genetic studies such as reporting mutations and polymorphisms (SNPs) associated with risk of gallbladder cancer
5. Systematic reviews and meta-analysis
6. Qualitative studies

Dutta EK, *et al. BMJ Open* 2022; 12:e056849. doi: 10.1136/bmjopen-2021-056849
