# Supplementary material for: Risk factors associated with gall bladder cancer in high incidence areas in India: a systematic review protocol
Source: BMJ Open. 2022 Mar 1;12(3):e056849. doi: 10.1136/bmjopen-2021-056849 (PMC8889324; doi:10.1136/bmjopen-2021-056849)
Supplement: Author's manuscript [file bmjopen-2021-056849.draft_revisions.pdf]

BMJ Open is committed to open peer review. As part of this commitment we make the peer review history of every article we publish publicly available.

When an article is published we post the peer reviewers' comments and the authors' responses online. We also post the versions of the paper that were used during peer review. These are the versions that the peer review comments apply to.

The versions of the paper that follow are the versions that were submitted during the peer review process. They are not the versions of record or the final published versions. They should not be cited or distributed as the published version of this manuscript.

BMJ Open is an open access journal and the full, final, typeset and author-corrected version of record of the manuscript is available on our site with no access controls, subscription charges or pay-per-view fees (http://bmjopen.bmj.com).

If you have any questions on BMJ Open's open peer review process please email info.bmjopen@bmj.com

BMJ Open

Risk factors associated with gall bladder cancer in high incident areas in India: a systematic review protocol

Journal:	*BMJ Open*
Manuscript ID	bmjopen-2021-056849
Article Type:	Protocol
Date Submitted by the Author:	02-Sep-2021
Complete List of Authors:	Dutta, Eliza; Indian Institutes of Public Health<br>Lewis, Melissa; Indian Institutes of Public Health<br>Albert, Sandra; Indian Institutes of Public Health
Keywords:	EPIDEMIOLOGY, Gastrointestinal tumours < ONCOLOGY, PUBLIC HEALTH

SCHOLARONE™
Manuscripts

Title: Risk factors associated with gall bladder cancer in high incident areas in India: a systematic review protocol

Eliza K Dutta, Melissa Glenda Lewis, Sandra Albert
Indian Institute of Public Health Shillong

Name, postal and e-mail address of the corresponding author:

Dr Eliza K Dutta
Indian Institute of Public Health Shillong
Pasteur hills, Lawmali,
Shillong, Meghalya-793001
India
+91-9823384186, Email: elizakdutta@gmail.com

Name and affiliations of co- authors:
Dr Melissa Glenda Lewis, Indian Institute of Public Health Shillong, Pasteur hills, Lawmali, Shillong, Meghalya-793001, India, +91-9880444159, Email: melissa.glenda1@gmail.com

Prof Sandra Albert, Indian Institute of Public Health Shillong, Pasteur hills, Lawmali, Shillong, Meghalya-793001, India,
+91-9108458517, Email: sandra.albert@phfi.org

Total Word count excluding title page, references, figures and tables: **3116**

Keywords: Gallbladder cancer, Risk-factors, High-risk zone, North India, North-East India

For peer review only

Risk factors associated with gall bladder cancer in high incident areas in India: a systematic review protocol

Abstract

Introduction: Gallbladder cancer (GBC) is a lethal form of malignancy of the gastro-intestinal tract with a unique geographical distribution. Cases are aggregated in the river basins of Ganga-Brahmaputra, in the north, east and north-east India, collectively termed as the 'high-risk' zone for GBC. Although some studies report high prevalence of typhoid infection linking with high burden of GBC in these regions, there is no systematic review of the factors associated with GBC in the high incident areas. To address this gap, we are conducting a systematic review to identify and organize the factors associated with GBC in the high-risk zone of India.

Methods and analysis: A systematic review of all observational studies that report a quantitative relationship between at-least one risk factor for GBC among adults (>18 years) in the high-risk zone in India will be conducted. The databases PubMed-MEDLINE, CINAHL, J-GATE, INDMED and Google scholar will be searched irrespective of any published date/year. This review will follow the Preferred Reporting Items for Systematic Reviews and Meta-Analyses (PRISMA) recommendations. The Primary outcome is gallbladder cancer. If data permits, meta-analysis will be performed. Two independent reviewers will independently screen the articles, extract the data, and assess the methodological quality of the studies.

Ethics and dissemination: As this will be a systematic review without human participants' involvement, there will be no requirement for ethics approval. Findings will be disseminated widely through peer-reviewed publication and media, e.g. conferences and symposia.

Registration number: CRD42021256673

Key words: Gallbladder cancer, Risk factors, North-East India, North India, High-risk zone

Strengths and limitations of this study:

1. The proposed systematic review addresses a gap by providing a comprehensive search on risk factors for gallbladder cancer from the high-risk zone of India, which reports the third-highest incidence in the world. Although sporadic narrative reviews exist, a systematic review on this topic is first from the region.
2. The review strictly follows the PRISMA guidelines.
3. This review is limited to observational studies and published in English.
4. There is a potential for low and inconsistent quality in the studies as we anticipate many small studies that are not adequately powered.

For peer review only - http://bmjopen.bmj.com/site/about/guidelines.xhtml

For peer review only

Introduction

Gallbladder cancer (GBC) is one of the most lethal forms of malignancy of the gastrointestinal tract with an overall survival <1 year.[1–4] Salient features of this cancer include a) non-specific presentation and asymptomatic progression,[5] and thus detection at a very late stage with poor prognosis b) unique geographical distribution[3,5] with cases aggregated in river basin zones such as the Ganga-Meghna-Brahmaputra (GMB) plain.[6] Population-based cancer registry (PBCR) data from India report the highest incidence in Kamrup district (Assam), 16.2 and 7.9 per 100,000 females and males,[7] respectively, only next to rates reported from Chilie and Korea.[8] The risk of incident GBC is seven times higher in the north and north-eastern states as compared to the south (ASR per 100,000 women: 8.6-17.1 in north/north-eastern PBCRs versus 0.7-4.1 in southern PBCRs).[3,7,9] Of 29 Indian PBCRs reporting GBC, the top five incident areas are situated in the north-east India (Kamrup, Cachar, and Dibrugarh districts in Assam, Papampure district in Arunachal Pradesh, Imphal west district in Manipur).[7] Evidence also indicates this risk remains same even after migration from 'high-risk' north and north-eastern zones to the 'low-risk' southern regions (OR:1.36, 95% 1.02-1.82).[5,9] This sustained risk, therefore, is not explained by the already studied risk factors such as gallstones, typhoid infection, etc. but could be due to sustained effects of exposure to environmental factors[4] such as pesticides,[10] heavy metals,[11,12] especially at an early time-point in life. Another possible explanation for sustained GBC risk in these populations could be genetic susceptibility. Familial Relative Risk of 3.15 with 23% heritability has been reported for GBC, indicating a significant contribution of genetic factors.[3]

Older age (> 50 years), female gender, obesity (BMI > 25 kg/m^2), chronic *Salmonella typhi* and, *Helicobacter bilis* infection, gallstone, especially with stone size > 3 cm and multiple stones are some of the established risk factors in the existing literature.[2,4,13,14] There is also evidence indicating moderate to a weak association between dietary patterns such as consumption of fried food, the interval between meals > 8 hours, exposure to carcinogen, coal/wood dust, and tobacco.[6,13,15] Reproductive factors such as multi-parity, use of oral contraceptive pills, early menarche, late menopause, too have shown associations with GBC, however, the strength of association remain unproven.[14,16] Although there are some evidence linking the prevalence of typhoid infection or exposure to arsenic, and geographical distribution of GBC in some parts of the world such as the Gangetic belt,[6,13] majority of these already established risk factors do not explain the unique geographical distribution of the disease, neither provides an understanding of the pattern of risk factors in the high incident regions, e.g. the north-eastern parts of the country, that reports the highest incidence of GBC.[7]

Lack of concrete evidence is attributable to the fact that most of the reviews conducted till date have been a) narrative reviews,[4,17] b) not specific to population rather comprising studies across the world,[4,18] and c) studies reporting associations based on post-surgical bio-specimen included along with epidemiological studies.[19] There is a paucity of systematic evidence of risk factors that will enable us to understand the unique distribution of GBC. There is also a scarcity of literature on the pattern of risk factors specific to high burden areas in the world. Existing systematic reviews[20] report association with single risk factors (e.g. size of gall stone, obesity) rather than providing evidence for population-specific risk factors.

As already stated, India especially the North and North-East India is one of the high-incidence hotspots for GBC[7], there is a paucity of systematically collated epidemiological evidence, that would help understand the pattern of risk factors for GBC, and in turn explain for high burden in these regions. The North-Eastern region of India is unique, home to different indigenous groups with unique socio-cultural and traditional behavioural practices. For example, tobacco use is an integral cultural practice in the region,[21–23] tracing back to

For peer review only - http://bmjopen.bmj.com/site/about/guidelines.xhtml

centuries, and the association between tobacco and the majority of cancers is well-established phenomenon.[24] This is supported by high incidence of cancers of the mouth/oral cavity, and nasopharynx in the region.[25] It is, therefore, necessary to study the patterns of risk factors in specific regions and populations to understand the distribution of a disease, GBC in this case. As of now, we do not have enough evidence on risk factors explaining the high burden of GBC in India or North-East India. We, therefore, propose to conduct a systematic review and meta-analysis of published literature to **identify GBC risk factors of the study population** (GMB belt in north, east, and north-east India), which would help identify and organize the factors associated with GBC in the high-risk zone of India, helping develop effective prevention strategies

Objective

To identify and list the risk factors that are associated with gallbladder cancer in high incidence areas in India.

Methods

Standards:

For the present protocol, we have followed the Preferred Reporting Items for Systematic Reviews and Meta-Analyses (PRISMA) guidelines.[26] This review is based on the methodology of the Cochrane handbook.[27] We will adhere to the PRISMA statement for the publication of the final review. We have registered the protocol International Prospective Register of Systematic Reviews (PROSPERO) database (No. CRD42021256673).[28]

Eligibility criteria

All Observational studies that include cohort, case-control, and cross-sectional that report a quantitative relationship between at least one risk factor with gall bladder cancer in the high incidence areas of India will be included. Articles that present only the incidence or prevalence of GBC will be excluded from this review. We will exclude studies that determined the effectiveness/efficacy of a treatment for GBC (randomised & non-randomised) unless they have assessed the risk factors. Reports from organisations, review papers, editorials, conference abstracts, research thesis, qualitative studies, interviews, case series, case reports, or studies that do not provide a quantitative relationship between the risk factor with gall bladder cancer will be excluded. This review will be limited to articles published in the English language irrespective of any published date/year. We will also exclude studies reporting associations based on bio-specimen without supporting epidemiological data.

Participants: Studies published on adults (>18 years) with confirmed gall bladder cancer in the high incidence areas in India will be included. In the present study, we define high incidence areas to be North, East, and North-East India, comprising the Ganga-Brahmaputra belt.

Disease: Adenocarcinoma of the gallbladder (ICD 10-C23) only will be included in this review.

Exposure: Risk factors associated with gallbladder cancer

Comparator: Participants not having gall bladder cancer will be the comparator group.

Outcomes: The present study aims to identify the risk factors associated with GBC. Hence, the outcome is gallbladder cancer.

For peer review only - http://bmjopen.bmj.com/site/about/guidelines.xhtml

Patient and public involvement: There will be no patient or public involvement in this study, as it's based on secondary data.

Information sources

A comprehensive literature search to identify all published and unpublished studies with no language restriction will be carried out. The electronic databases included in the search are PubMed-MEDLINE, CINAHL, J-GATE (meta-search engine), INDMED(regional database), and Google Scholar. For each electronic database, a search strategy specific to that database will be developed. The search strategy for PubMed is available in the supplementary file 1.

Searching other resources

We will follow a three-stage selection process for the final inclusion of studies in the review. In the first stage, two reviewers will assess each title for its appropriateness for inclusion in the review. If found inappropriate then the title will be rejected, and all other titles will be moved to the second stage of selection. In the second stage, abstracts of titles will be obtained, and two reviewers will independently scrutinize all such abstracts. Here again, whenever both reviewers agreed to reject, such studies will be rejected, and the remaining studies will be obtained and reviewed by two authors independently. If both reviewers agreed to accept then those studies will be included, articles will be rejected when both reviewers agreed to reject. In case of disagreement between reviewers, a third reviewer will arbitrate the selection process. A PRISMA chart will be used to present the flow of the selection process.

Data Extraction

Data will be extracted using a predesigned and pretested proforma. The proforma will include the following, year of publication, authors, region, state, study design, study setting, information on the type participants, age group, gender, sampling technique, sample size, examined risk factor, and its relationship with GBC (example incidence ratio/prevalence ratio/ mortality ratio/odds ratio/relative risk/hazards ratio). Information on all the confounding variables used for analysis, unadjusted and adjusted estimates will be extracted. Studies will be divided into two groups - adjusted or not adjusted for confounding factors. Data will be extracted from full-text articles by one reviewer and will be reviewed by a second reviewer. Disagreements if any will be discussed will be drawn.

Risk of Bias

ROBINS-I (risk of bias in non-randomised studies of interventions) tool[29] will be used for assessing the risk of bias for the final set of articles on case-control, cohort, and cross-sectional studies. We will treat studies that do not adjust risk factors for potentially confounding variables as "high" risk of bias. Two reviewers will independently assess the risk of bias and conflicts will be resolved through consultation with a third reviewer. Cochrane risk of bias tool[30] will be used if interventional studies are obtained, although not likely.

Data synthesis

Firstly, we will provide a list of all confirmed risk factors mentioned in the included studies in a narrative format. A detailed summary of all the included studies will include information on authors, type of study design, participants, age, gender, region, sample size, the risk factor with a measure of association, primary findings including limitations. Secondly, an evaluation will be conducted if it is appropriate to perform a meta-analysis to assess the relationship of risk factors with GBC. Meta-analysis with random effects model will be performed if there is a similarity in terms of the participants, study design, and risk factors. The results will be expressed in relative risk, prevalence ratio, and odds ratio with 95% confidence intervals when

For peer review only - http://bmjopen.bmj.com/site/about/guidelines.xhtml

appropriate. Forest plots, I^2 statistic, Chi^2 test, and Tau^2 will be used to measure and assess heterogeneity among the included studies in each analysis. Meta-regression will be used to investigate heterogeneity if needed. An attempt will be made to contact study authors if data is inadequate or missing and a record will be maintained on the amount of missing data with reasons. An assessment for publication bias will be made by creating a funnel plot only if there are at least 10 studies in the meta-analysis.

Analysis of subgroups or subsets: If enough data is available, subgroup analysis will be performed by gender, age, and region.

Current study status: Review ongoing

Ethics and dissemination: To our information, this will be the first study to synthesize results on the relationship between risk factors and gallbladder cancer risk. The findings from the review will be helpful for future studies/reviews to compare the pattern of risk factors from other geographies/populations. These findings will be summarised and presented at conferences and through publication in a peer-reviewed journal**.** This study involves analysis of data from published literature and does not involve individual-level identifiable data. Given this, there were no privacy concerns that required ethical approval.

Acknowledgments: We acknowledge the India Alliance DBT-Wellcome Trust for funding this work under the ‘Early Career Fellowship’ in clinical research and public health. We also acknowledge the Indian Institute of Public Health, Shillong for providing necessary infrastructural support.

Author affiliations: ED, ML, and SA are affiliated to Indian Institute of Public Health, Shillong

Contributorship statement: ED conceptualized, designed the protocol, drafted the initial and reviewed the protocol, ML planned data extraction and statistical analysis and, SA defined the clinical concepts and reviewed the final protocol

Competing interests: None declared

Funding: India Alliance DBT-Wellcome Trust ‘Early Career Fellowship’ in clinical research and public health, grant no. IA/CPHE/19/1/504604

Bibliography

1 Bourtros C, Gary M, Baldwin K, Somasundar P. Gallbladder cancer: past, present and an uncertain future. *Surg Oncol* 2012; **21**: e183-91.
2 Randi G, Franceschi S, Vecchia CL. Gallbladder cancer worldwide: Geographical distribution and risk factors. *International Journal of Cancer* 2006; **118**: 1591–602.
3 Mhatre S, Wang Z, Nagrani R, *et al.* Common genetic variation and risk of gallbladder cancer in India: a case-control genome-wide association study. *Lancet Oncol* 2017; **18**: 535–44.
4 Hundal R, Shaffer EA. Gallbladder cancer: epidemiology and outcome. *Clin Epidemiol* 2014; **6**: 99–109.
5 Mehrotra R, Tulsyan S, Hussain S, *et al.* Genetic landscape of gallbladder cancer: Global overview. *Mutat Res* 2018; **778**: 61–71.
6 Unisa S, Jagannath P, Dhir V, Khandelwal C, Sarangi L, Roy TK. Population-based study to estimate prevalence and determine risk factors of gallbladder diseases in the rural Gangetic basin of North India. *HPB (Oxford)* 2011; **13**: 117–25.
7 Indian Council of Medical Research. National cancer registry programme: population-based cancer registries. http://www.pbcrindia.org/ (accessed March 11, 2018).
8 Ferlay J, Soerjomataram I, Dikshit R, *et al.* Cancer incidence and mortality worldwide: sources, methods and major patterns in GLOBOCAN 2012. *Int J Cancer* 2015; **136**: E359-386.
9 Mhatre SS, Nagrani RT, Budukh A, *et al.* Place of birth and risk of gallbladder cancer in India. *Indian J Cancer* 2016; **53**: 304–8.
10 Shukla VK, Rastogi AN, Adukia TK, Raizada RB, Reddy DC, Singh S. Organochlorine pesticides in carcinoma of the gallbladder: a case-control study. *Eur J Cancer Prev* 2001; **10**: 153–6.

For peer review only - http://bmjopen.bmj.com/site/about/guidelines.xhtml

11 Basu S, Singh M. Heavy and trace metals in carcinoma of the gallbladder. https://pubmed.ncbi.nlm.nih.gov/23942528/ (accessed March 5, 2021).
12 Mondal B, Maulik D, Mandal M, Sarkar GN, Sengupta S, Ghosh D. Analysis of Carcinogenic Heavy Metals in Gallstones and its Role in Gallbladder Carcinogenesis. *J Gastrointest Cancer* 2017; **48**: 361–8.
13 Jain K, Sreenivas V, Velpandian T, Kapil U, Garg PK. Risk factors for gallbladder cancer: a case-control study. *Int J Cancer* 2013; **132**: 1660–6.
14 Pandey M, Shukla VK. Lifestyle, parity, menstrual and reproductive factors and risk of gallbladder cancer. *Eur J Cancer Prev* 2003; **12**: 269–72.
15 Dwivedi AND, Jain S, Dixit R. Gall bladder carcinoma: Aggressive malignancy with protean loco-regional and distant spread. *World J Clin Cases* 2015; **3**: 231–44.
16 Shukla VK, Chauhan VS, Mishra RN, Basu S. Lifestyle, reproductive factors and risk of gallbladder cancer. *Singapore Med J* 2008; **49**: 912–5.
17 Dutta U, Bush N, Kalsi D, Popli P, Kapoor VK. Epidemiology of gallbladder cancer in India. *Chinese Clinical Oncology* 2019; **8**: 4–4.
18 Nemunaitis JM, Brown-Glabeman U, Soares H, *et al.* Gallbladder cancer: review of a rare orphan gastrointestinal cancer with a focus on populations of New Mexico. *BMC Cancer* 2018; **18**: 665.
19 Chhabra D, Oda K, Jagannath P, Utsunomiya H, Takekoshi S, Nimura Y. Chronic heavy metal exposure and gallbladder cancer risk in India, a comparative study with Japan. *Asian Pac J Cancer Prev* 2012; **13**: 187–90.
20 Mahdavifar N, Mohammadian M, Salehiniya H. Gallbladder cancer in the world: epidemiology, incidence, mortality and risk factors. ; : 7.
21 Global Adult Tobacco Survey. https://www.who.int/teams/noncommunicable-diseases/surveillance/systems-tools/global-adult-tobacco-survey (accessed Aug 6, 2021).
22 Goyal G, Bhagawati BT. Knowledge, Attitude and Practice of Chewing Gutka, Areca Nut, Snuff and Tobacco Smoking Among the Young Population in the Northern India Population. *Asian Pac J Cancer Prev* 2016; **17**: 4813–8.
23 Kwai: The Taste of Meghalaya. https://www.outlookindia.com/newswire/story/kwai-the-taste-of-meghalaya/678657 (accessed Aug 6, 2021).
24 Asthana S, Patil RS, Labani S. Tobacco-related cancers in India: A review of incidence reported from population-based cancer registries. *Indian J Med Paediatr Oncol* 2016; **37**: 152–7.
25 Dhillon PK, Mathur P, Nandakumar A, *et al.* The burden of cancers and their variations across the states of India: the Global Burden of Disease Study 1990–2016. *The Lancet Oncology* 2018; **19**: 1289–306.
26 Page MJ, McKenzie JE, Bossuyt PM, *et al.* The PRISMA 2020 statement: an updated guideline for reporting systematic reviews. *BMJ* 2021; : n71.
27 Cochrane Handbook for Systematic Reviews of Interventions. https://training.cochrane.org/handbook (accessed Aug 6, 2021).
28 Dutta E, Lewis M, Albert S. Risk factors associated with gall bladder cancer in high incident areas in India. PROSPERO 2021 CRD42021256673. https://www.crd.york.ac.uk/prospero/display_record.php?ID=CRD42021256673.
29 ROBINS-I tool. https://methods.cochrane.org/methods-cochrane/robins-i-tool (accessed Aug 6, 2021).
30 Higgins JPT, Altman DG, Gøtzsche PC, *et al.* The Cochrane Collaboration’s tool for assessing risk of bias in randomised trials. *BMJ* 2011; **343**: d5928.

For peer review only

For peer review only - http://bmjopen.bmj.com/site/about/guidelines.xhtml

Review title: Risk factors associated with gall bladder cancer in high incident areas in India

Appendix 1

Keywords as per Population Concept Context (PCC):

Population: Adults (>18 years) with confirmed gall bladder cancer

Concept: Risk factors (exposures)

Context: High incidence areas in India defined as the North, East and North-East India, comprising the Ganga-Brahmaputra belt.

S.No	Theme	Search strategy (Key words)
1	Gallbladder Cancer	"Gallbladder cancer*" OR "Gall bladder cancer"<br>"gallbladder neoplasms"[MeSH Terms] OR "Gall bladder neoplasm*"<br>"Gallbladder carcinoma*" OR "Gall bladder carcinoma*"<br>"Carcinoma of gallbladder" OR "Carcinoma of gall bladder"<br>"Gallbladder tumor*" OR "Gall bladder tumor*"<br>"Cancer of gallbladder" OR "Cancer of gall bladder"
2	Risk factors	'Risk factor*' OR 'epidemiological factor*' OR 'association' OR 'correlat*' OR relationship
3	India	India*[pl]

Inclusion criteria:
Studies

1. Reporting association (positive or negative or no) with at-least one risk factors for gallbladder cancer mortality, incidence, hazards, survival, causation
2. States indicative of geographical region within north and northeastern India.
3. Results based on biospecimens only from Indian patients residing in India
4. Conducted after year 1990
5. limited to English language
6. Studies indicative of north and North East/Northern or north eastern India.

Exclusion criteria-
Studies

1. Measuring only prevalence/incidence of gallbladder cancer
2. Experimental studies measuring efficacy of therapeutic/surgical interventions
3. Conducted on Indian population* living outside India
4. Genetic studies such as reporting mutations and polymorphisms (SNPs) associated with risk of gallbladder cancer
5. Systematic reviews and meta-analysis
6. Qualitative studies

For peer review only - http://bmjopen.bmj.com/site/about/guidelines.xhtml

PRISMA-P (Preferred Reporting Items for Systematic review and Meta-Analysis Protocols) 2015 checklist: recommended items to address in a systematic review protocol*

Section and topic	Item No	Checklist item	Location (Page)
**ADMINISTRATIVE INFORMATION**			
Title:			
Identification	1a	Identify the report as a protocol of a systematic review	2
Update	1b	If the protocol is for an update of a previous systematic review, identify as such	NA
Registration	2	If registered, provide the name of the registry (such as PROSPERO) and registration number	2
Authors:			
Contact	3a	Provide name, institutional affiliation, e-mail address of all protocol authors; provide physical mailing address of corresponding author	6
Contributions	3b	Describe contributions of protocol authors and identify the guarantor of the review	6
Amendments	4	If the protocol represents an amendment of a previously completed or published protocol, identify as such and list changes; otherwise, state plan for documenting important protocol amendments	NA
Support:			
Sources	5a	Indicate sources of financial or other support for the review	6
Sponsor	5b	Provide name for the review funder and/or sponsor	6
Role of sponsor or funder	5c	Describe roles of funder(s), sponsor(s), and/or institution(s), if any, in developing the protocol	6
**INTRODUCTION**			
Rationale	6	Describe the rationale for the review in the context of what is already known	3
Objectives	7	Provide an explicit statement of the question(s) the review will address with reference to participants, interventions, comparators, and outcomes (PICO)	4
**METHODS**			
Eligibility criteria	8	Specify the study characteristics (such as PICO, study design, setting, time frame) and report characteristics (such as years considered, language, publication status) to be used as criteria for eligibility for the review	4
Information sources	9	Describe all intended information sources (such as electronic databases, contact with study authors, trial registers or other grey literature sources) with planned dates of coverage	5
Search strategy	10	Present draft of search strategy to be used for at least one electronic database, including planned limits, such that it could be repeated	Supplementary file 1

For peer review only

Study records:			
Data management	11a	Describe the mechanism(s) that will be used to manage records and data throughout the review	5
Selection process	11b	State the process that will be used for selecting studies (such as two independent reviewers) through each phase of the review (that is, screening, eligibility and inclusion in meta-analysis)	5
Data collection process	11c	Describe planned method of extracting data from reports (such as piloting forms, done independently, in duplicate), any processes for obtaining and confirming data from investigators	5
Data items	12	List and define all variables for which data will be sought (such as PICO items, funding sources), any pre-planned data assumptions and simplifications	4,5
Outcomes and prioritization	13	List and define all outcomes for which data will be sought, including prioritization of main and additional outcomes, with rationale	5
Risk of bias in individual studies	14	Describe anticipated methods for assessing risk of bias of individual studies, including whether this will be done at the outcome or study level, or both; state how this information will be used in data synthesis	5
Data synthesis	15a	Describe criteria under which study data will be quantitatively synthesised	5
	15b	If data are appropriate for quantitative synthesis, describe planned summary measures, methods of handling data and methods of combining data from studies, including any planned exploration of consistency (such as I^2, Kendall's τ)	5
	15c	Describe any proposed additional analyses (such as sensitivity or subgroup analyses, meta-regression)	5,6
	15d	If quantitative synthesis is not appropriate, describe the type of summary planned	NA
Meta-bias(es)	16	Specify any planned assessment of meta-bias(es) (such as publication bias across studies, selective reporting within studies)	6
Confidence in cumulative evidence	17	Describe how the strength of the body of evidence will be assessed (such as GRADE)	NA

*** It is strongly recommended that this checklist be read in conjunction with the PRISMA-P Explanation and Elaboration (cite when available) for important clarification on the items. Amendments to a review protocol should be tracked and dated. The copyright for PRISMA-P (including checklist) is held by the PRISMA-P Group and is distributed under a Creative Commons Attribution Licence 4.0.**

From: Shamseer L, Moher D, Clarke M, Ghersi D, Liberati A, Petticrew M, Shekelle P, Stewart L, PRISMA-P Group. Preferred reporting items for systematic review and meta-analysis protocols (PRISMA-P) 2015: elaboration and explanation. BMJ. 2015 Jan 2;349(jan02 1):g7647.

For peer review only

BMJ Open

Risk factors associated with gall bladder cancer in high incident areas in India: a systematic review protocol

Journal:	*BMJ Open*
Manuscript ID	bmjopen-2021-056849.R1
Article Type:	Protocol
Date Submitted by the Author:	13-Dec-2021
Complete List of Authors:	Dutta, Eliza; Indian Institutes of Public Health<br>Lewis, Melissa; Indian Institutes of Public Health<br>Albert, Sandra; Indian Institutes of Public Health
**Primary Subject Heading**:	Public health
Secondary Subject Heading:	Oncology, Epidemiology
Keywords:	EPIDEMIOLOGY, Gastrointestinal tumours < ONCOLOGY, PUBLIC HEALTH

SCHOLARONE™
Manuscripts

Title: Risk factors associated with gall bladder cancer in high incident areas in India: a systematic review protocol

Eliza K Dutta, Melissa Glenda Lewis, Sandra Albert
Indian Institute of Public Health Shillong

Name, postal and e-mail address of the corresponding author:

Dr Eliza K Dutta
Indian Institute of Public Health Shillong
Pasteur hills, Lawmali,
Shillong, Meghalya-793001
India
+91-9823384186, Email: elizakdutta@gmail.com

Name and affiliations of co- authors:
Dr Melissa Glenda Lewis, Indian Institute of Public Health Shillong, Pasteur hills, Lawmali, Shillong, Meghalya-793001, India, +91-9880444159, Email: melissa.glenda1@gmail.com

Prof Sandra Albert, Indian Institute of Public Health Shillong, Pasteur hills, Lawmali, Shillong, Meghalya-793001, India,
+91-9108458517, Email: sandra.albert@phfi.org

Total Word count excluding title page, references, figures and tables: **2344**

Keywords: Gallbladder cancer, Risk-factors, High-risk zone, North India, North-East India

1

For peer review only

Risk factors associated with gall bladder cancer in high incident areas in India: a systematic review protocol

Abstract

Introduction: Gallbladder cancer (GBC) is a lethal form of malignancy of the gastro-intestinal tract with a unique geographical distribution. Cases are aggregated in the river basins of Ganga-Brahmaputra, in the north, east and north-east India, collectively termed as the 'high-risk' zone for GBC. Although some studies report high prevalence of typhoid infection linking with high burden of GBC in these regions, there is no systematic review of the factors associated with GBC in the high incident areas. To address this gap, we are conducting a systematic review to identify and organize the factors associated with GBC in the high-risk zone of India.

Methods and analysis: A systematic review of all observational studies that report a quantitative relationship between at-least one risk factor for GBC among adults (>18 years) in the high-risk zone in India will be conducted. The databases PubMed-MEDLINE, CINAHL, EMBASE, Web of Science, Scopus, OpenGrey and Google scholar will be searched published in English and after 1990.. This review will follow the Preferred Reporting Items for Systematic Reviews and Meta-Analyses (PRISMA) recommendations. The Primary outcome is gallbladder cancer. If data permits, meta-analysis will be performed. Two independent reviewers will independently screen the articles, extract the data, and assess the methodological quality of the studies.

Ethics and dissemination: As this will be a systematic review without human participants' involvement, there will be no requirement for ethics approval. Findings will be disseminated widely through peer-reviewed publication and media, e.g. conferences and symposia.

Registration number: CRD42021256673

Key words: Gallbladder cancer, Risk factors, North-East India, North India, High-risk zone

Strengths and limitations of this study:

1. The proposed systematic review addresses a gap by providing a comprehensive search on risk factors for gallbladder cancer from the high-risk zone of India, which reports the third-highest incidence in the world. Although sporadic narrative reviews exist, a systematic review on this topic is first from the region.
2. The review strictly follows the PRISMA guidelines.
3. This review is limited to observational studies and published in English.
4. There is a potential for low and inconsistent quality in the studies as we anticipate many small studies that are not adequately powered.

For peer review only - http://bmjopen.bmj.com/site/about/guidelines.xhtml

Introduction

Gallbladder cancer (GBC) is one of the most lethal forms of malignancy of the gastrointestinal tract with an overall survival <1 year.[1–4] Salient features of this cancer include a) non-specific presentation and asymptomatic progression,[5] and thus detection at a very late stage with poor prognosis b) unique geographical distribution[3,5] with cases aggregated in river basin zones such as the Ganga-Meghna-Brahmaputra (GMB) plain.[6] Population-based cancer registry (PBCR) data from India report the highest incidence in Kamrup district (Assam), 16.2 and 7.9 per 100,000 females and males,[7] respectively, only next to rates reported from Chilie and Korea.[8] The risk of incident GBC is seven times higher in the north and north-eastern states as compared to the south (ASR per 100,000 women: 8.6-17.1 in north/north-eastern PBCRs versus 0.7-4.1 in southern PBCRs).[3,7,9] Of 29 Indian PBCRs reporting GBC, the top five incident areas are situated in the north-east India (Kamrup, Cachar, and Dibrugarh districts in Assam, Papampure district in Arunachal Pradesh, Imphal west district in Manipur).[7] Evidence also indicates this risk remains same even after migration from 'high-risk' north and north-eastern zones to the 'low-risk' southern regions (OR:1.36, 95% 1.02-1.82).[5,9] This sustained risk, therefore, is not explained by the already studied risk factors such as gallstones, typhoid infection, etc. but could be due to sustained effects of exposure to environmental factors[4] such as pesticides,[10] heavy metals,[11,12] especially at an early time-point in life. Another possible explanation for sustained GBC risk in these populations could be genetic susceptibility. Familial Relative Risk of 3.15 with 23% heritability has been reported for GBC, indicating a significant contribution of genetic factors.[3]

Older age (> 50 years), female gender, obesity (BMI > 25 kg/m^2), chronic *Salmonella typhi* and, *Helicobacter bilis* infection, gallstone, especially with stone size > 3 cm and multiple stones are some of the established risk factors in the existing literature.[2,4,13,14] There is also evidence indicating moderate to a weak association between dietary patterns such as consumption of fried food, the interval between meals > 8 hours, exposure to carcinogen, coal/wood dust, and tobacco.[6,13,15] Reproductive factors such as multi-parity, use of oral contraceptive pills, early menarche, late menopause, too have shown associations with GBC, however, the strength of association remain unproven.[14,16] Although there are some evidence linking the prevalence of typhoid infection or exposure to arsenic, and geographical distribution of GBC in some parts of the world such as the Gangetic belt,[6,13] majority of these already established risk factors do not explain the unique geographical distribution of the disease, neither provides an understanding of the pattern of risk factors in the high incident regions, e.g. the north-eastern parts of the country, that reports the highest incidence of GBC.[7]

Lack of concrete evidence is attributable to the fact that most of the reviews conducted till date have been a) narrative reviews,[4,17] b) not specific to population rather comprising studies across the world,[4,18] and c) studies reporting associations based on post-surgical bio-specimen included along with epidemiological studies.[19] There is a paucity of systematic evidence of risk factors that will enable us to understand the unique distribution of GBC. There is also a scarcity of literature on the pattern of risk factors specific to high burden areas in the world. Existing systematic reviews[20] report association with single risk factors (e.g. size of gall stone, obesity) rather than providing evidence for population-specific risk factors.

As already stated, India especially the North and North-East India is one of the high-incidence hotspots for GBC[7], there is a paucity of systematically collated epidemiological evidence, that would help understand the pattern of risk factors for GBC, and in turn explain for high burden in these regions. The North-Eastern region of India is unique, home to different indigenous groups with unique socio-cultural and traditional behavioural practices. For example, tobacco use is an integral cultural practice in the region,[21–23] tracing back to

For peer review only - http://bmjopen.bmj.com/site/about/guidelines.xhtml

centuries, and the association between tobacco and the majority of cancers is well-established phenomenon.[24] This is supported by high incidence of cancers of the mouth/oral cavity, and nasopharynx in the region.[25] It is, therefore, necessary to study the patterns of risk factors in specific regions and populations to understand the distribution of a disease, GBC in this case. As of now, we do not have enough evidence on risk factors explaining the high burden of GBC in India or North-East India. We, therefore, propose to conduct a systematic review and meta-analysis of published literature to **identify GBC risk factors of the study population** (GMB belt in north, east, and north-east India), which would help identify and organize the factors associated with GBC in the high-risk zone of India, helping develop effective prevention strategies

Objective

To identify and list the risk factors that are associated with gallbladder cancer in high incidence areas in India.

Methods

Standards:

For the present protocol, we have followed the Preferred Reporting Items for Systematic Reviews and Meta-Analyses (PRISMA) guidelines.[26] This review is based on the methodology of the Cochrane handbook.[27] We will adhere to the PRISMA statement for the publication of the final review. We have registered the protocol International Prospective Register of Systematic Reviews (PROSPERO) database (No. CRD42021256673).[28]

Eligibility criteria

All Observational studies that include cohort, case-control, and cross-sectional that report a quantitative relationship between at least one risk factor with gall bladder cancer in the high incidence areas of India will be included. Articles that present only the incidence or prevalence of GBC will be excluded from this review. We will exclude studies that determined the effectiveness/efficacy of a treatment for GBC (randomised & non-randomised) unless they have assessed the risk factors. Reports from organisations, review papers, editorials, conference abstracts, research thesis, qualitative studies, interviews, case series, case reports, or studies that do not provide a quantitative relationship between the risk factor with gall bladder cancer will be excluded. This review will be limited to articles published in the English language published after 1990. We will also exclude studies reporting genetic associations with GBC.

Participants: Studies published on adults (>18 years) with confirmed gall bladder cancer in the high incidence areas in India will be included. In the present study, we define high incidence areas to be North, East, and North-East India, comprising the Ganga-Brahmaputra belt.

Disease: Adenocarcinoma of the gallbladder (ICD 10-C23) only will be included in this review.

Exposure: Risk factors associated with gallbladder cancer

Comparator: Participants not having gall bladder cancer will be the comparator group.

Outcomes: The present study aims to identify the risk factors associated with GBC. Hence, the outcome is gallbladder cancer.

For peer review only - http://bmjopen.bmj.com/site/about/guidelines.xhtml

Patient and public involvement: There will be no patient or public involvement in this study, as it's based on secondary data.

Information sources

A comprehensive literature search to identify all published and unpublished studies with no language restriction will be carried out. The electronic databases included in the search are PubMed-MEDLINE, EMBASE, Web of science, Scopus, CINAHLplus, OpenGrey. and Google Scholar. For each electronic database, a search strategy specific to that database will be developed. The search strategy for all the above databases are available in the supplementary file 1.

Searching other resources

We will follow a three-stage selection process for the final inclusion of studies in the review. In the first stage, two reviewers will assess each title for its appropriateness for inclusion in the review. If found inappropriate then the title will be rejected, and all other titles will be moved to the second stage of selection. In the second stage, abstracts of titles will be obtained, and two reviewers will independently scrutinize all such abstracts. Here again, whenever both reviewers agreed to reject, such studies will be rejected, and the remaining studies will be obtained and reviewed by two authors independently. If both reviewers agreed to accept then those studies will be included, articles will be rejected when both reviewers agreed to reject. In case of disagreement between reviewers, a third reviewer will arbitrate the selection process. A PRISMA chart will be used to present the flow of the selection process.

Data Extraction

Data will be extracted using a predesigned and pretested proforma. The proforma will include the following, year of publication, authors, region, state, study design, study setting, information on the type participants, age group, gender, sampling technique, sample size, examined risk factor along withtechniques used to measure each risk factor and its relationship with GBC (example incidence ratio/prevalence ratio/ mortality ratio/odds ratio/relative risk/hazards ratio). Information on all the confounding variables used for analysis, unadjusted and adjusted estimates will be extracted. Studies will be divided into two groups - adjusted or not adjusted for confounding factors. Data will be extracted from full-text articles by one reviewer and will be reviewed by a second reviewer. Disagreements if any will be discussed will be drawn.

Risk of Bias

ROBINS-I (risk of bias in non-randomised studies of interventions) tool[29] will be used for assessing the risk of bias for the final set of articles on case-control, cohort, and cross-sectional studies. We will treat studies that do not adjust risk factors for potentially confounding variables as "high" risk of bias. Two reviewers will independently assess the risk of bias and conflicts will be resolved through consultation with a third reviewer. Cochrane risk of bias tool[30] will be used if interventional studies are obtained, although not likely.

Data synthesis

Firstly, we will provide a list of all confirmed risk factors mentioned in the included studies in a narrative format. A detailed summary of all the included studies will include information on authors, type of study design, participants, age, gender, region, sample size, the risk factor with a measure of association, primary findings including limitations. Secondly, an evaluation will be conducted if it is appropriate to perform a meta-analysis to assess the relationship of risk factors with GBC. Meta-analysis with random effects model will be performed if there is a

For peer review only - http://bmjopen.bmj.com/site/about/guidelines.xhtml

similarity in terms of the participants, study design, and risk factors. The results will be expressed in relative risk, prevalence ratio, and odds ratio with 95% confidence intervals when appropriate. Forest plots, I^2 statistic, Chi^2 test, and Tau^2 will be used to measure and assess heterogeneity among the included studies in each analysis. Meta-regression will be used to investigate heterogeneity if needed. An attempt will be made to contact study authors if data is inadequate or missing and a record will be maintained on the amount of missing data with reasons. An assessment for publication bias will be made by creating a funnel plot only if there are at least 10 studies in the meta-analysis.

Analysis of subgroups or subsets: If enough data are available, subgroup analysis will be performed by demographic, anthropometric factors, nutritional factors, reproductive factors, exposure to environmental factors, typhoid/enteric fever, dietary/cooking practices and geographical factors.

Current study status: Review ongoing.

Start date of the review: June 2021

Anticipated end-date of review: September 2022

Ethics and dissemination: To our information, this will be the first study to synthesize results on the relationship between risk factors and gallbladder cancer risk. The findings from the review will be helpful for future studies/reviews to compare the pattern of risk factors from other geographies/populations. These findings will be summarised and presented at conferences and through publication in a peer-reviewed journal. This study involves analysis of data from published literature and does not involve individual-level identifiable data. Given this, there were no privacy concerns that required ethical approval.

Acknowledgments: We acknowledge the India Alliance DBT-Wellcome Trust for funding this work under the ‘Early Career Fellowship’ in clinical research and public health. We also acknowledge the Indian Institute of Public Health, Shillong for providing necessary infrastructural support.

Author affiliations: ED, ML, and SA are affiliated to Indian Institute of Public Health, Shillong

Contributorship statement: ED conceptualized, designed the protocol, drafted the initial and reviewed the protocol, ML planned data extraction and statistical analysis and, SA defined the clinical concepts and reviewed the final protocol

Competing interests: None declared

Funding: India Alliance DBT-Wellcome Trust ‘Early Career Fellowship’ in clinical research and public health, grant no. IA/CPHE/19/1/504604

Bibliography

1 Bourtros C, Gary M, Baldwin K, Somasundar P. Gallbladder cancer: past, present and an uncertain future. *Surg Oncol* 2012; **21**: e183-91.

2 Randi G, Franceschi S, Vecchia CL. Gallbladder cancer worldwide: Geographical distribution and risk factors. *International Journal of Cancer* 2006; **118**: 1591–602.

3 Mhatre S, Wang Z, Nagrani R, *et al.* Common genetic variation and risk of gallbladder cancer in India: a case-control genome-wide association study. *Lancet Oncol* 2017; **18**: 535–44.

4 Hundal R, Shaffer EA. Gallbladder cancer: epidemiology and outcome. *Clin Epidemiol* 2014; **6**: 99–109.

5 Mehrotra R, Tulsyan S, Hussain S, *et al.* Genetic landscape of gallbladder cancer: Global overview. *Mutat Res* 2018; **778**: 61–71.

For peer review only - http://bmjopen.bmj.com/site/about/guidelines.xhtml

6 Unisa S, Jagannath P, Dhir V, Khandelwal C, Sarangi L, Roy TK. Population-based study to estimate prevalence and determine risk factors of gallbladder diseases in the rural Gangetic basin of North India. *HPB (Oxford)* 2011; **13**: 117–25.

7 Indian Council of Medical Research. National cancer registry programme: population-based cancer registries. http://www.pbcrindia.org/ (accessed March 11, 2018).

8 Ferlay J, Soerjomataram I, Dikshit R, *et al.* Cancer incidence and mortality worldwide: sources, methods and major patterns in GLOBOCAN 2012. *Int J Cancer* 2015; **136**: E359-386.

9 Mhatre SS, Nagrani RT, Budukh A, *et al.* Place of birth and risk of gallbladder cancer in India. *Indian J Cancer* 2016; **53**: 304–8.

10 Shukla VK, Rastogi AN, Adukia TK, Raizada RB, Reddy DC, Singh S. Organochlorine pesticides in carcinoma of the gallbladder: a case-control study. *Eur J Cancer Prev* 2001; **10**: 153–6.

11 Basu S, Singh M. Heavy and trace metals in carcinoma of the gallbladder. https://pubmed.ncbi.nlm.nih.gov/23942528/ (accessed March 5, 2021).

12 Mondal B, Maulik D, Mandal M, Sarkar GN, Sengupta S, Ghosh D. Analysis of Carcinogenic Heavy Metals in Gallstones and its Role in Gallbladder Carcinogenesis. *J Gastrointest Cancer* 2017; **48**: 361–8.

13 Jain K, Sreenivas V, Velpandian T, Kapil U, Garg PK. Risk factors for gallbladder cancer: a case-control study. *Int J Cancer* 2013; **132**: 1660–6.

14 Pandey M, Shukla VK. Lifestyle, parity, menstrual and reproductive factors and risk of gallbladder cancer. *Eur J Cancer Prev* 2003; **12**: 269–72.

15 Dwivedi AND, Jain S, Dixit R. Gall bladder carcinoma: Aggressive malignancy with protean loco-regional and distant spread. *World J Clin Cases* 2015; **3**: 231–44.

16 Shukla VK, Chauhan VS, Mishra RN, Basu S. Lifestyle, reproductive factors and risk of gallbladder cancer. *Singapore Med J* 2008; **49**: 912–5.

17 Dutta U, Bush N, Kalsi D, Popli P, Kapoor VK. Epidemiology of gallbladder cancer in India. *Chinese Clinical Oncology* 2019; **8**: 4–4.

18 Nemunaitis JM, Brown-Glabeman U, Soares H, *et al.* Gallbladder cancer: review of a rare orphan gastrointestinal cancer with a focus on populations of New Mexico. *BMC Cancer* 2018; **18**: 665.

19 Chhabra D, Oda K, Jagannath P, Utsunomiya H, Takekoshi S, Nimura Y. Chronic heavy metal exposure and gallbladder cancer risk in India, a comparative study with Japan. *Asian Pac J Cancer Prev* 2012; **13**: 187–90.

20 Mahdavifar N, Mohammadian M, Salehiniya H. Gallbladder cancer in the world: epidemiology, incidence, mortality and risk factors. ; : 7.

21 Global Adult Tobacco Survey. https://www.who.int/teams/noncommunicable-diseases/surveillance/systems-tools/global-adult-tobacco-survey (accessed Aug 6, 2021).

22 Goyal G, Bhagawati BT. Knowledge, Attitude and Practice of Chewing Gutka, Areca Nut, Snuff and Tobacco Smoking Among the Young Population in the Northern India Population. *Asian Pac J Cancer Prev* 2016; **17**: 4813–8.

23 Kwai: The Taste of Meghalaya. https://www.outlookindia.com/newswire/story/kwai-the-taste-of-meghalaya/678657 (accessed Aug 6, 2021).

24 Asthana S, Patil RS, Labani S. Tobacco-related cancers in India: A review of incidence reported from population-based cancer registries. *Indian J Med Paediatr Oncol* 2016; **37**: 152–7.

25 Dhillon PK, Mathur P, Nandakumar A, *et al.* The burden of cancers and their variations across the states of India: the Global Burden of Disease Study 1990–2016. *The Lancet Oncology* 2018; **19**: 1289–306.

26 Page MJ, McKenzie JE, Bossuyt PM, *et al.* The PRISMA 2020 statement: an updated guideline for reporting systematic reviews. *BMJ* 2021; : n71.

27 Cochrane Handbook for Systematic Reviews of Interventions. https://training.cochrane.org/handbook (accessed Aug 6, 2021).

28 Dutta E, Lewis M, Albert S. Risk factors associated with gall bladder cancer in high incident areas in India. PROSPERO 2021 CRD42021256673. https://www.crd.york.ac.uk/prospero/display_record.php?ID=CRD42021256673.

29 ROBINS-I tool. https://methods.cochrane.org/methods-cochrane/robins-i-tool (accessed Aug 6, 2021).

30 Higgins JPT, Altman DG, Gøtzsche PC, *et al.* The Cochrane Collaboration's tool for assessing risk of bias in randomised trials. *BMJ* 2011; **343**: d5928.

For peer review only - http://bmjopen.bmj.com/site/about/guidelines.xhtml

Review title: Risk factors associated with gall bladder cancer in high incident areas in India
Supplementary file:1
PCC Statement: Population: Adults (>18 years) with confirmed gall bladder cancer
Concept: Risk factors (exposures)
Context: High incidence areas in India defined as the North, East and North-East India, comprising the Ganga-Brahmaputra belt.
Search strategy used for each database:

1. **PUBMED**

#1-Domain 1
"gallbladder cancer*"[All Fields] OR "Gall bladder cancer"[All Fields] OR "Gallbladder neoplasms"[MeSH Terms] OR "gall bladder neoplasm*"[All Fields] OR "gallbladder carcinoma*"[All Fields] OR "gall bladder carcinoma*"[All Fields] OR "Carcinoma of gallbladder"[All Fields] OR "gallbladder tumor*"[All Fields] OR "gall bladder tumor*"[All Fields] OR "Cancer of gallbladder"[All Fields]
#**2-Domain 2**
"risk factor*"[All Fields] OR "epidemiological factor*"[All Fields] OR "associate"[All Fields] OR "associated"[All Fields] OR "associates"[All Fields] OR "associating"[All Fields] OR "association"[MeSH Terms] OR "association"[All Fields] OR "associations"[All Fields] OR "correlat*"[All Fields] OR "relation*"[All Fields]
#3- Domain 3
"india"[MeSH Terms] OR "india"[All Fields] OR "india s"[All Fields] OR "indias"[All Fields]
#1 AND #2 AND #3
Final Search
("gallbladder cancer*"[All Fields] OR "Gall bladder cancer"[All Fields] OR "gallbladder neoplasms"[MeSH Terms] OR "gall bladder neoplasm*"[All Fields] OR "gallbladder carcinoma*"[All Fields] OR "gall bladder carcinoma*"[All Fields] OR "Carcinoma of gallbladder"[All Fields] OR "gallbladder tumor*"[All Fields] OR "gall bladder tumor*"[All Fields] OR "Cancer of gallbladder"[All Fields]) AND ("risk factor*"[All Fields] OR "epidemiological factor*"[All Fields] OR "correlat*"[All Fields] OR "associat*"[All Fields] OR "relation*"[All Fields] OR "association"[MeSH Terms]) AND ("india"[MeSH Terms] OR "india"[All Fields] OR "india s"[All Fields] OR "indias"[All Fields])"associating"[All Fields] OR "association"[MeSH Terms] OR "association"[All Fields] OR "associations"[All Fields] OR "correlat*"[All Fields] OR "relation*"[All Fields])
Total number of titles: 384

2. **EMBASE**

Domain 1:
("gallbladder cancer*" or "Gall bladder cancer" or "Gallbladder neoplasms" or "gall bladder neoplasm*" or "gallbladder carcinoma*" or "gall bladder carcinoma*" or "Carcinoma of gallbladder" or "gallbladder tumor*"or "gall bladder tumor*" or "Cancer of gallbladder").af.
Domain 2:
('risk factor*' or 'epidemiological factor*' or correlat* or relation* or associat*).af
Domain 3:
(india* or India*).af.
Final search:
1 and 2 and 3
Number of titles: 942
Set filters to English language, human and year1990 onwards
Total number of titles: 885

For peer review only - http://bmjopen.bmj.com/site/about/guidelines.xhtml

3. Web of Science

Domain 1

((((((((((ALL=("gallbladder cancer*")) OR ALL=("Gall bladder cancer"))) OR ALL=("Gallbladder neoplasms")) OR ALL=("gall bladder neoplasm*")) OR ALL=("gallbladder carcinoma*")) OR ALL=("gall bladder carcinoma*")) OR ALL=("Carcinoma of gallbladder")) OR ALL=("gallbladder tumor*")) OR ALL=("gall bladder tumor*")) OR ALL=("Cancer of gallbladder")

Domain 2

((((ALL=("risk factor*")) OR ALL=("epidemiological factor*")) OR ALL=("associat*")) OR ALL=(correlat*)) OR ALL=(relation*)

Domain 3

(ALL=(india*)) OR ALL=(India*)

Final search:

((#1) AND #2) AND #3

Total number of titles: 373

4. **SCOPUS**

Domain 1

"gallbladder AND *cancer*"* OR *"Gall bladder cancer"* OR *"Gallbladder neoplasms"* OR *"gall bladder ne OR oplasm*"* OR *"gallbladder carcinoma*"* OR *"gall bladder carcinoma*"* OR *"Carcinoma of gallbladder"* OR *"gallbladder tumor*"* OR *"gall bladder tumor*"* OR *"Cancer of gallbladder"*

Domain 2

'risk AND *factor*'* OR *epidemiological* AND *factor*'* OR *correlat** OR *relation** OR *associat* ** Domain 3

*india** OR *india**

Final search:

(*'risk* AND *factor*'* OR *'epidemiological* AND *factor*'* OR *correlat** OR *relation** OR *associ at**) AND (*"gallbladder* AND *cancer*"* OR *"Gall bladder cancer"* OR *"Gallbladder neoplasms"* OR *"gall bladder ne OR oplasm*"* OR *"gallbladder carcinoma*"* OR *"gall bladder carcinoma*"* OR *"Carcinoma of gallbladder"* OR *"gallbladder tumor*"* OR *"gall bladder tumor*"* OR *"Cancer of gallbladder"**) AND (*india** OR *india**) AND (EXCLUDE (PUBYEAR , *1989*) OR EXCLUDE (PUBYEAR , *1980*) OR EXCLUDE (PUBYEAR , *1978*) OR EXCLUDE (PUBYEAR , *1976*))

Total number of titles: 212

5. CINAHL plus (EBSCO)

Domain 1

"gallbladder AND cancer*" OR "Gall bladder cancer" OR "Gallbladder neoplasms" OR "gall bladder ne OR oplasm*" OR "gallbladder carcinoma*" OR "gall bladder carcinoma*" OR "Carcinoma of gallbladder" OR "gallbladder tumor*" OR "gall bladder tumor*" OR "Cancer of gallbladder"

Domain 2

'risk AND factor*' OR 'epidemiological AND factor*' OR correlat* OR relation* OR associat*

Domain 3

india* OR India*

Final search:

S1 AND S2 AND S3

Total number of titles: 36

For peer review only - http://bmjopen.bmj.com/site/about/guidelines.xhtml

6. **Open Grey**

("gallbladder AND cancer*" OR "Gall bladder cancer" OR "Gallbladder neoplasms" OR "gall bladder ne OR oplasm*" OR "gallbladder carcinoma*" OR "gall bladder carcinoma*" OR "Carcinoma of gallbladder" OR "gallbladder tumor*" OR "gall bladder tumor*" OR "Cancer of gallbladder") AND ('risk AND factor*' OR 'epidemiological AND factor*' OR correlat* OR relation* OR associat*) AND india* OR India*

Total number of title = 1

7. **GoogleScholar**

("gallbladder cancer" OR "Gall bladder cancer" OR "Gallbladder neoplasms" OR "gall bladder neoplasm" OR "gallbladder carcinoma" OR "gall bladder carcinoma" OR "Carcinoma of gallbladder" OR "gallbladder tumor" OR "gall bladder tumor" OR "Cancer of gallbladder") AND ('risk facto" OR epidemiological OR factor OR correlation OR relation OR relationship OR association OR associated) AND India

Number of titles = 146

Added filter for articles between 1990-2021

Total number of titles = 93

Inclusion criteria:

Studies

1. Reporting association (positive or negative or no) with at-least one risk factors for gallbladder cancer mortality, incidence, hazards, survival, causation
2. Conducted among India patients, residing in India
3. Results based on biospecimens only from Indian patients residing in India
4. Conducted after year 1990
5. limited to English language
6. Studies not mentioning 'India' in their title or abstract but study setting indicative of geographical region within India

Exclusion criteria:

Studies

1. Measuring only prevalence/incidence of gallbladder cancer
2. Experimental studies measuring efficacy of therapeutic/surgical interventions
3. Conducted on Indian population* living outside India
4. Genetic studies such as reporting mutations and polymorphisms (SNPs) associated with risk of gallbladder cancer
5. Systematic reviews and meta-analysis
6. Qualitative studies

For peer review only - http://bmjopen.bmj.com/site/about/guidelines.xhtml

PRISMA-P (Preferred Reporting Items for Systematic review and Meta-Analysis Protocols) 2015 checklist: recommended items to address in a systematic review protocol*

Section and topic	Item No	Checklist item	Location (Page)
**ADMINISTRATIVE INFORMATION**			
Title:			
Identification	1a	Identify the report as a protocol of a systematic review	2
Update	1b	If the protocol is for an update of a previous systematic review, identify as such	NA
Registration	2	If registered, provide the name of the registry (such as PROSPERO) and registration number	2
Authors:			
Contact	3a	Provide name, institutional affiliation, e-mail address of all protocol authors; provide physical mailing address of corresponding author	6
Contributions	3b	Describe contributions of protocol authors and identify the guarantor of the review	6
Amendments	4	If the protocol represents an amendment of a previously completed or published protocol, identify as such and list changes; otherwise, state plan for documenting important protocol amendments	NA
Support:			
Sources	5a	Indicate sources of financial or other support for the review	6
Sponsor	5b	Provide name for the review funder and/or sponsor	6
Role of sponsor or funder	5c	Describe roles of funder(s), sponsor(s), and/or institution(s), if any, in developing the protocol	6
**INTRODUCTION**			
Rationale	6	Describe the rationale for the review in the context of what is already known	3
Objectives	7	Provide an explicit statement of the question(s) the review will address with reference to participants, interventions, comparators, and outcomes (PICO)	4
**METHODS**			
Eligibility criteria	8	Specify the study characteristics (such as PICO, study design, setting, time frame) and report characteristics (such as years considered, language, publication status) to be used as criteria for eligibility for the review	4
Information sources	9	Describe all intended information sources (such as electronic databases, contact with study authors, trial registers or other grey literature sources) with planned dates of coverage	5
Search strategy	10	Present draft of search strategy to be used for at least one electronic database, including planned limits, such that it could be repeated	Supplementary file 1

For peer review only

Study records:			
Data management	11a	Describe the mechanism(s) that will be used to manage records and data throughout the review	5
Selection process	11b	State the process that will be used for selecting studies (such as two independent reviewers) through each phase of the review (that is, screening, eligibility and inclusion in meta-analysis)	5
Data collection process	11c	Describe planned method of extracting data from reports (such as piloting forms, done independently, in duplicate), any processes for obtaining and confirming data from investigators	5
Data items	12	List and define all variables for which data will be sought (such as PICO items, funding sources), any pre-planned data assumptions and simplifications	4,5
Outcomes and prioritization	13	List and define all outcomes for which data will be sought, including prioritization of main and additional outcomes, with rationale	5
Risk of bias in individual studies	14	Describe anticipated methods for assessing risk of bias of individual studies, including whether this will be done at the outcome or study level, or both; state how this information will be used in data synthesis	5
Data synthesis	15a	Describe criteria under which study data will be quantitatively synthesised	5
	15b	If data are appropriate for quantitative synthesis, describe planned summary measures, methods of handling data and methods of combining data from studies, including any planned exploration of consistency (such as I^2, Kendall's τ)	5
	15c	Describe any proposed additional analyses (such as sensitivity or subgroup analyses, meta-regression)	5,6
	15d	If quantitative synthesis is not appropriate, describe the type of summary planned	NA
Meta-bias(es)	16	Specify any planned assessment of meta-bias(es) (such as publication bias across studies, selective reporting within studies)	6
Confidence in cumulative evidence	17	Describe how the strength of the body of evidence will be assessed (such as GRADE)	NA

*** It is strongly recommended that this checklist be read in conjunction with the PRISMA-P Explanation and Elaboration (cite when available) for important clarification on the items. Amendments to a review protocol should be tracked and dated. The copyright for PRISMA-P (including checklist) is held by the PRISMA-P Group and is distributed under a Creative Commons Attribution Licence 4.0.**

From: Shamseer L, Moher D, Clarke M, Ghersi D, Liberati A, Petticrew M, Shekelle P, Stewart L, PRISMA-P Group. Preferred reporting items for systematic review and meta-analysis protocols (PRISMA-P) 2015: elaboration and explanation. BMJ. 2015 Jan 2;349(jan02 1):g7647.

For peer review only
